# Supplementary material for: Faster Short-Chain Fatty Acid Absorption from the Cecum Following Polydextrose Ingestion Increases the Salivary Immunoglobulin A Flow Rate in Rats
Source: Nutrients. 2020 Jun 11;12(6):1745. doi: 10.3390/nu12061745 (PMC7353249; doi:10.3390/nu12061745)
Supplement: Supplementary file 1 [file nutrients-12-01745-s001.zip › Revision Supplementary Materials.docx]

| **Ingredient** | **Control** | **PDX** |
| --- | --- | --- |
| Sucrose | 700 | 660 |
| Casein | 200 | 200 |
| Corn oil | 50.0 | 50.0 |
| Mineral mixture (AIN-76) * | 35.0 | 35.0 |
| Vitamin mixture (AIN-76) ^†^ | 10.0 | 10.0 |
| dl-Methionine | 3.00 | 3.00 |
| Choline bitartrate | 2.00 | 2.00 |
| PDX | 0 | 40.0 |
| Total | 1000 | 1000 |

**Table S1.** Control and polydextrose (PDX) diets (g/kg) composition

*Mineral mixture content (AIN-76) (g/kg): calcium phosphate 500, sodium chloride 74.0,

potassium citrate 220, potassium sulfate 52.0, magnesium oxide 24.0, magnesium carbonate

3.50, ferric citrate 6.00, zinc carbonate 1.60, cupric carbonate 0.300, potassium iodate 0.0100,

sodium selenite 0.0100, chromium potassium sulfate 0.550, and sucrose 118.

^†^Vitamin mixture content (AIN-76) (mg/kg): thiamin 600, riboflavin 600, pyridoxin 700, niacin

300, calcium pantothenate 160, folic acid 200, biotin 200, cyanocobalamin 10.0, retinol 24,000,

cholecalciferol 2.50, tocotrienols 5,000, menadione 5.00, and sucrose 979,000.


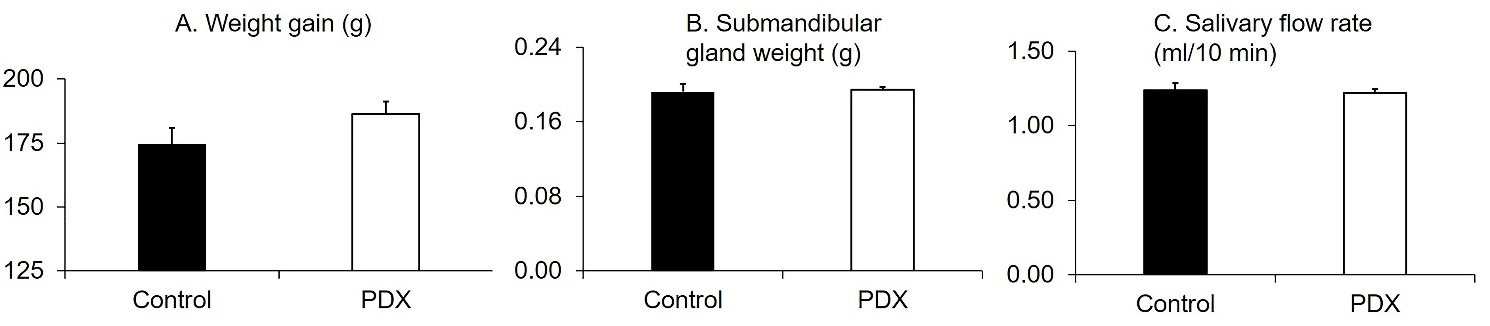


**Figure S1.** Effects of polydextrose (PDX) addition on the weight gain (A), weight of the submandibular gland (B), and salivary flow rate (C). n = 6 per group. Data are expressed as means (histogram bars) and SEM (error bars). We found no significant differences in the body weight gain, mean weight of left and right submandibular glands, and the saliva flow rate for 10 min after the 28-day feeding period between the two groups (p = 0.2, 0.8, and 0.7, respectively, Welch's *t*-test).
